# The Detection of Food Additives Using a Fluorescence Indicator Based on 6– p–Toluidinylnaphthalence-2-sulfonate and Cationic Pillar[6]arene

**DOI:** 10.3389/fchem.2022.925881

**Published:** 2022-05-31

**Authors:** Qunpeng Duan, Yibo Xing, Kainan Guo

**Affiliations:** School of Chemical and Printing-dyeing Engineering, Henan University of Engineering, Zhengzhou, China

**Keywords:** pillararene, host-guest complex, fluorescence, indicator displacement assay, food additives

## Abstract

The current study investigated host-guest complexation in 6-p-toluidinylnaphthalene-2-sulfonate (**TNS**), a fluorescence probe used to investigate hydrophobic regions that contain the water-soluble cationic pillar[6]arene (**CP6**). After complexation with **CP6**, the fluorescence intensity of **TNS** was significantly increased. The decreases in the fluorescence intensity of the **TNS**•**CP6** complex when phenolic food-additives are added have been used in indicator displacement assays to detect food additives in the water.

## Introduction

Fluorescent indicator displacement (FID) assays make use of fluorescent indicators and emission phenomena to detect important analytes by transiting different receptors to universal optical sensors. FID assays can bind to a wide variety of target molecules (Rather et al., 2021; Sedgwick et al., 2021). With the advancement of host-guest chemistry, macrocyclic host-based FID assays have garnered widespread attention for their potential application in the field of analytical testing, and several significant research results have been generated in recent years (Dsouza et al., 2011; Ghale et al., 2014; Cao et al., 2019; Jiang et al., 2020). Macrocyclic hosts, primarily cyclodextrins (Crini, et al., 2014; Pal, et al., 2015), calixarenes (Koh, et al., 1996; Hennig, et al., 2007; Guo, et al., 2014; Zheng, et al., 2018), cucurbiturils (Praetorius, et al., 2008; Florea, et al., 2011; Barrow, et al., 2015; Sonzini, et al., 2017), and pillararenes (Wang, et al., 2014; Bojtár, et al., 2015; Bojtár, et al., 2016; Hua, et al., 2016; Bojtár, et al., 2017; Hua, et al., 2018; Cai, et al., 2021; Wu, et al., 2021), are widely used as fluorescent probes in the majority of the FID-based sensing systems.

Phenolic food additives have been widely used in the food industry for their significant antioxidant, antimicrobial, and flavor-enhancing properties (Vinson, et al., 2012; Zhang, et al., 2014). Whereas the insolubility of food additives in water and their long-term stability contribute to their excessive use, ultimately resulting in their accumulation and negative effects on the biosphere (Tobacman, et al., 2001; Savjani, et al., 2012). Encapsulating small-molecule food-additives in non-toxic, water-soluble macrocyclic hosts improves their bioavailability and solubility by regulating their physical and chemical properties (Munin, et al., 2011). 2-Hydroxypropyl β-cyclodextrin (HP-β-CD) is one representative example, with a binding affinity of ∼ 10^2^ M^−1^ to food-additives (Pal, et al., 2016). It is critical to investigate artificial receptors with extremely high affinity for food additives to improve sensitivity and detection efficiency in compound detection.

**SCHEME 1:**
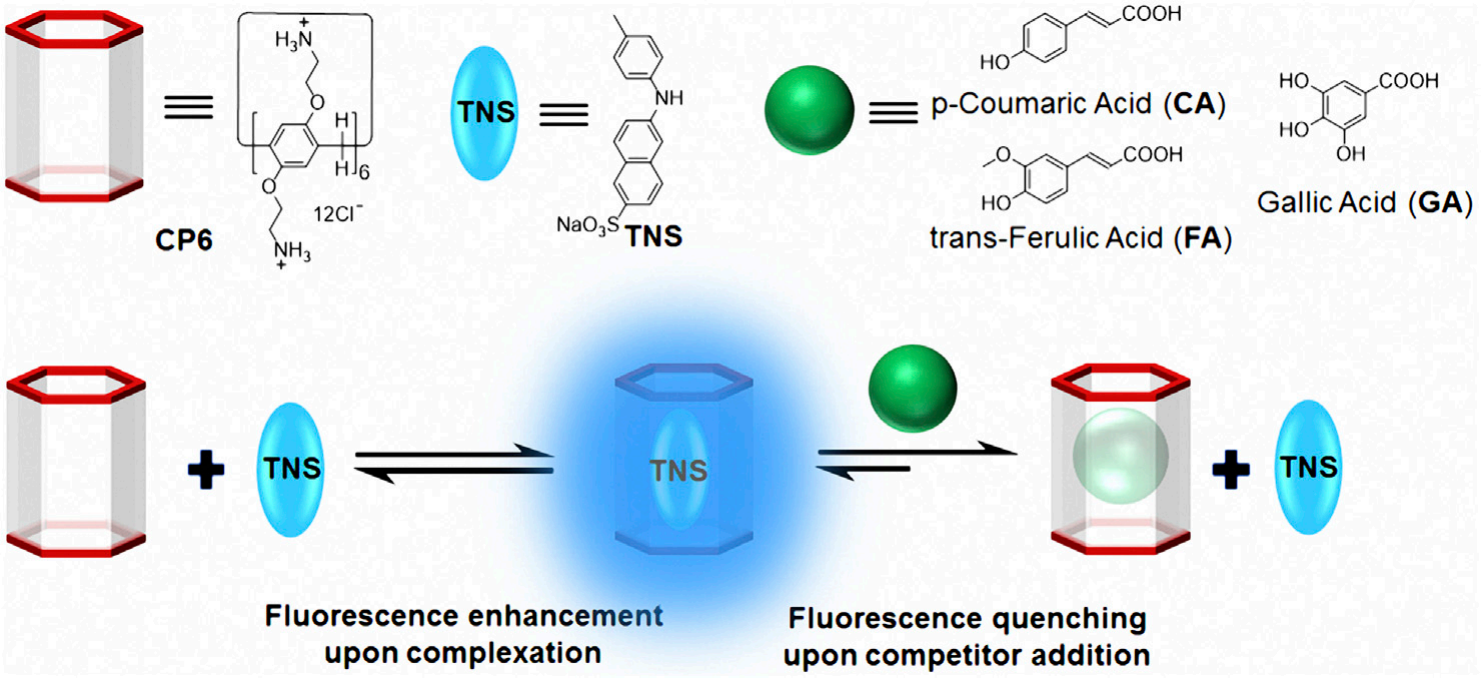
Chemical structures and cartoon representations of **CP6**, **TNS**, **CA**, **FA**, and **GA**, as well as an illustration of the procedure for fluorescence indicator displacement.

We developed a new FID assay with a water-soluble cationic pillar[6]arene (**CP6**) for the detection of three important phenolic food additives, namely p-coumaric acid (**CA**), trans-ferulic acid (**FA**), and gallic acid (**GA**). Because of its enhanced fluorescence in non-polar environments, the widely used fluorescent probe, 6-p-toluidinylnaphthalene-2-sulfonate (Dotsikas, et al., 2000) (**TNS** in Scheme 1), was used as the fluorescent indicator in our FID system. Due to the complexation of **TNS** and **CP6**, we use an FID strategy to perform sensitive fluorescence detection on **CA**, **FA**, and **GA**.

## Materials and Methods

The reagents used were marketable and applied directly without further purification. **CP6** (Duan et al., 2019) was synthesized by following the known procedures. Nuclear magnetic resonance (NMR) spectra were obtained using the Bruker Avance III HD 400 spectrometer with the deuterated solvent as the lock and the residual solvent as the internal reference. Fluorescence spectra were obtained by using the Agilent Cary Eclipse fluorescence spectrophotometer. To prevent the dilution effect during titration, **CP6** stock solutions were produced using the same **TNS** solution. The measurement was repeated three times for each experiment. Displacement assays for **CA**, **FA**, and **GA** were performed at pH 6.8 with **CP6** at varying concentrations of **CA**, **FA**, and **GA**, respectively. All the experiments were conducted at room temperature (298 K).

## Results and Discussion

### Fluorescent Probe 6-p-Toluidinylnaphthalene-2-Sulfonate Complexed With Cationic Pillar[6]Arene

**FIGURE 1:**
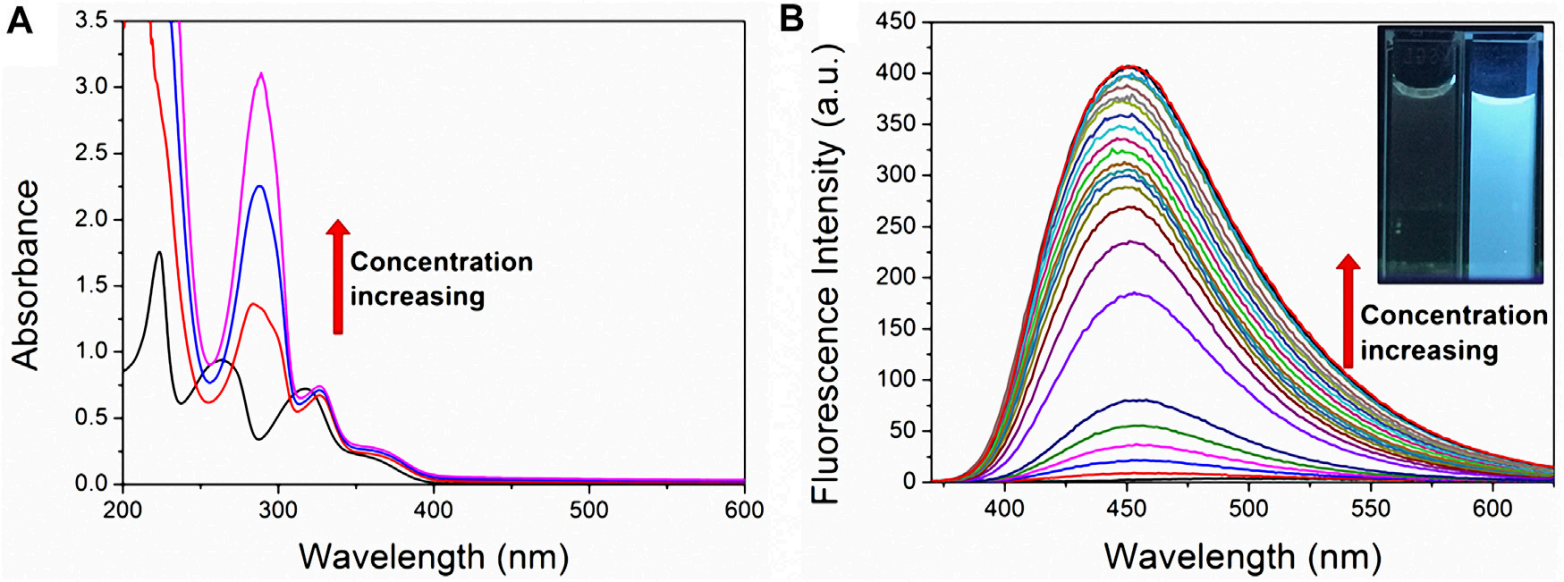
UV-vis and fluorescence titration on **TNS** with **CP6** in PBS (pH 6.8). **(A)** Absorption spectra (20 µM **TNS**, 0–6.5 equiv. **CP6**) **(B)** fluorescence spectra (20 µM **TNS**, 0–2.6 equiv. **CP6**, *λ*_ex_ = 318 nm). The inset illustrates enhanced fluorescence in water when excited at 365 nm with a UV lamp set to 298 K.

UV-vis absorption spectroscopy was used to confirm the host-guest complexation of fluorescent probe **TNS** with **CP6**. Following successive additions of **CP6** to the phosphate-buffered solution (PBS) of **TNS** at pH 6.8, hyperchromic effects at the maximum absorption wavelengths of 223, 263, and 318 nm occurred with a significant bathochromic shift (Figure 1A). The variations appeared to be greater than those when α-CD (Nishijo et al., 1992) or β-CD (Nishijo et al., 1995; Dotsikas et al., 2000) addition was used. The results indicate that **TNS** can form a stable complex with **CP6**. Additionally, a fluorescence titration on **TNS** with an increased **CP6** concentration was performed in PBS with a pH of 6.8 at room temperature. According to Figure 1B, as the concentration of **CP6** increased, a significant increase in fluorescence intensity was observed, along with a shift in the fluorescence maximum to shorter wavelengths. The results indicate that **TNS** molecules exist in a hydrophobic environment. Encapsulating **TNS** in **CP6** protects it from solvent collisions while also providing a distinct local environment for **TNS** in terms of polarity, which significantly enhances (approximately 400 times) fluorescence intensity. Additionally, the significant pale blue fluorescence was evident in UV light (the inset of Figure 1B).

**FIGURE 2:**
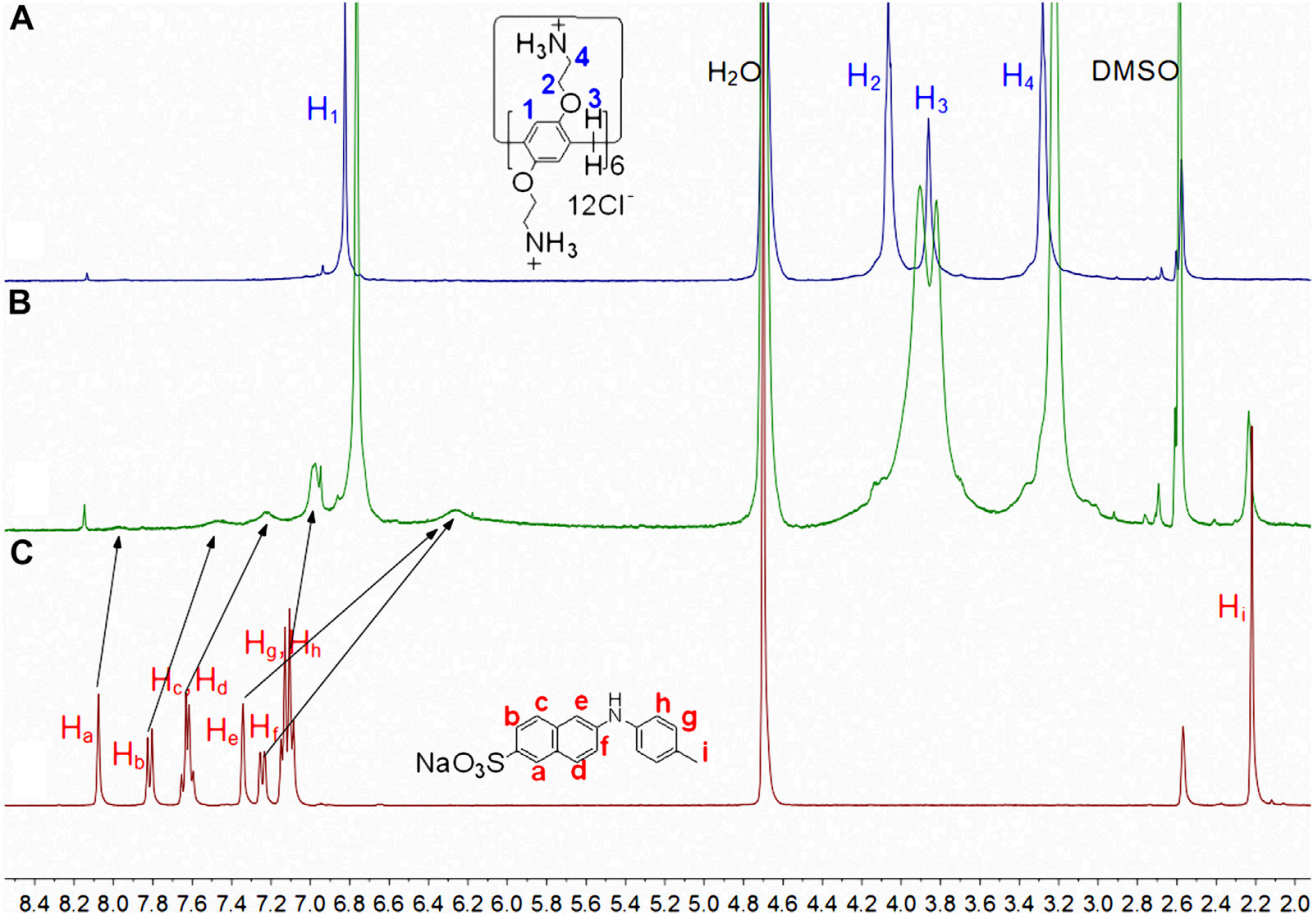
Partial ^1^H NMR spectra (400 MHz, D_2_O:DMSO-*d*_6_ = 3:1, 298 K) for **(A)** 5 mM **CP6**, **(B)** 5 mM **CP6** and 15 mM **TNS**, **(C)** 15 mM **TNS**.

^1^H NMR tests were used to investigate the host-guest complexation. Because the solubility of the complex in neat D_2_O was insufficient to reach the mM level, DMSO-*d*_6_ cosolvents were added. As illustrated in Figure 2, the naphthyl proton signals of **TNS** in the inclusion complex underwent varying degrees of upward shifts. The largest shift occurred in the direction away from the sulfonate group, whereas the smallest shift occurred in the direction toward the sulfonate group. Proton signal variations in the methylphenyl group are insignificant, indicating that this group may be located outside the cavity. When combined with the protons’ shift and broadening in the sulfonate-naphthyl group, it is concluded that the fluorescence probe molecule is partially in the **CP6** cavity, where the shielding effects of the aromatic host produce the characteristic signal broadening (Li et al., 2010). Additionally, the 2D ROESY data (Supplementary Figure S1) establish a correlation between the naphthyl protons (H_a-f_) in the entrapped **TNS** and the aromatic proton H_1_ in **CP6**, revealing the interpenetrated geometry.

To quantify the binding of **TNS** to **CP6**, the association constant (*K*_a_) was determined to be (4.51 ± 0.90) × 10^5^ M^−1^ using titration tests and a non-linear curve-fitting to the fluorescence spectra (Supplementary Figure S4). The complex formed by **CP6** and **TNS** had a 1:1 binding stoichiometry (Supplementary Figure S3A). We deduced that the complex formed between **CP6** and **TNS** in aqueous solution as a result of multiple electrostatic interactions between the cationic ammonium groups on **CP6** and the sulfonate anion on **TNS**, hydrophobic interactions, and π-π stacking interactions between the benzene rings on host **CP6** and naphthalene ring on guest **TNS**. The cooperativity of these non-covalent interactions is attributed to the binding affinity in the host-guest system.

### Detection of Food-Additives Using Fluorescent Indicator

**FIGURE 3:**
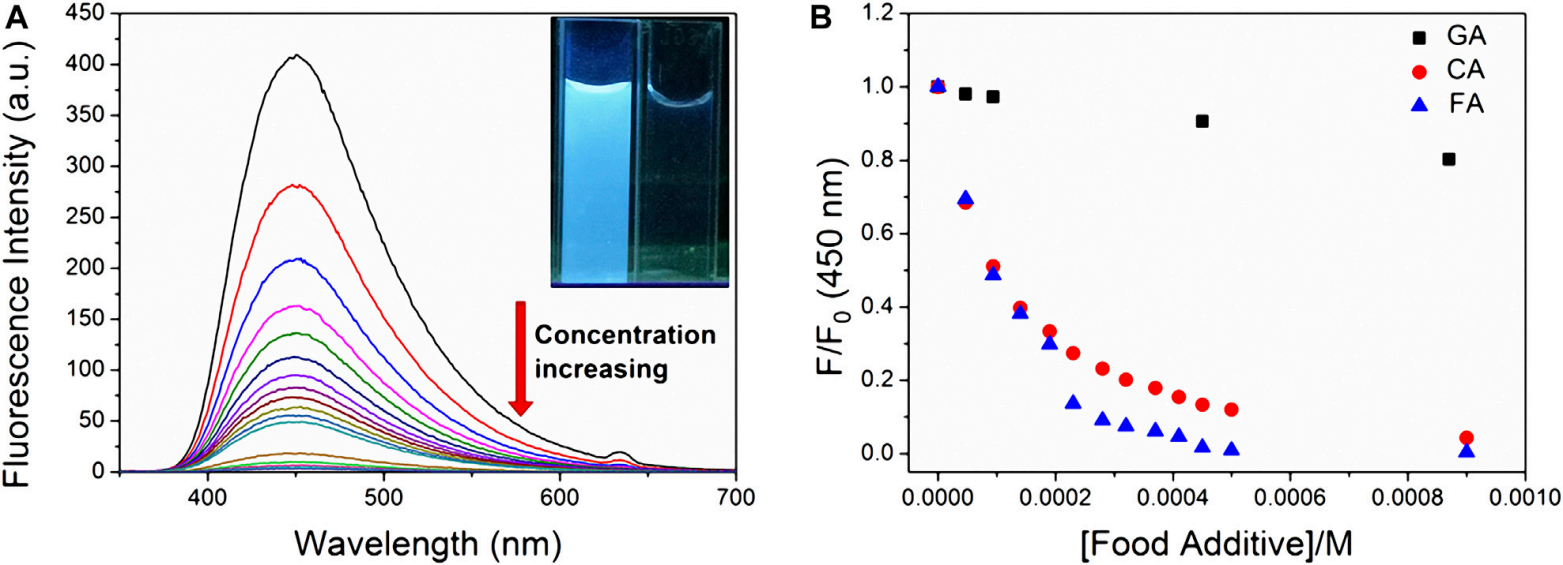
Displacement of the fluorescent indicator **CP6•TNS** by various food additives in PBS (pH 6.8). **(A)** Fluorescence spectra of **CP6•TNS** (20 µM **TNS**, 48 µM **CP6**, *λ*_ex_ = 318 nm) upon addition of **CA** (0–2.3 mM). The inset reveals fluorescence quenching in water at excitation of 365 nm under the UV lamp at 298 K. **(B)** Fluorescence intensity changes at 450 nm of **CP6•TNS** (20 µM **TNS**, 48 µM **CP6**, *λ*_ex_ = 318 nm) upon addition of **CA**, **FA**, and **GA** in different concentrations.

**TNS** complexed with **CP6** exhibits a significant fluorescence response, allowing it to be used for FID detection. The binding affinities of **CP6** to **CA**, **FA**, and **GA** were determined in this study using FID detection (Scheme 1), and the fluorescent indicator **TNS** was first reversibly bound to the receptor **CP6**. The solution was then added with a weakly fluorescent or non-fluorescent analyte, which competitively displaced the highly fluorescent **TNS** from the indicator **CP6** cavity, altering the optical signal (You et al., 2015). Notably, titration of a preformed **TNS**•**CP6** complex with increasing concentrations of competitor food additive molecules results in a reversal of the fluorescence intensity, which is used to determine the competitor molecules’ binding affinity. Competitive displacement was used to investigate the binding of three major phenolic food-additives, namely **CA**, **FA**, and **GA**, to **CP6**. Figure 3A illustrates a typical fluorescence displacement titration with **CA** as a strong competitor. The quenching of fluorescence in the presence of **CA** was easily observed with the naked eye using a simple UV-lamp (the inset of Figure 3A).

To avoid the effect of changes in pH and dilution on the displacement assay, the pH of complex and food-additive solutions was set to 6.8, and the concentrations of **TNS** and **CP6** in food-additive solutions were kept constant. We used that previously reported competitive binding formula to fit the reduced fluorescence intensities at the band maximum against the concentration of competitor food additives (Bakirci et al., 2006). Using **TNS**•**CP6** as the reporter pair, we determined the association constants (*K*_a_) for **CA**, **FA**, and **GA** to be (1.24 ± 0.29) × 10^4^ M^−1^, (1.19 ± 0.16) × 10^4^ M^−1^, and (2.78 ± 0.18) × 10^2^ M^−1^ (Supplementary Figures S5–S7), respectively. Except for **GA**, the binding affinities are approximately two orders of magnitude greater than those of previously studied HP-*β*-CD to the other two food additives, which are around 10^2^ M^−1^ (Pal et al., 2016).

Additionally, the observed fluorescence response can also be used to quantify **CA**, **FA**, and **GA**. The fluorescence intensity plots increase linearly as the **CA**, **FA**, and **GA** concentrations increase (Supplementary Figure S8), respectively. 0.047–2.3 mM, 0.047–0.14 mM, and 0.047–2.5 mM were the linear ranges. The results indicate that the limit of detection (LOD) values was 0.012, 0.08, and 0.17 µM, respectively, using a 3σ/slope method (MacDougall et al., 1980).

NMR research with **GA**, **CA**, and **FA** was used to determine the complexation of food additives. Supplementary Figure S9 illustrates the ^1^H NMR spectra for **GA** in the presence of **CP6**. As illustrated in the figure, shielding caused a shift in the benzene proton signal of **GA**, conclusively confirming the inclusion complex between **CP6** and **GA**. Additionally, the 2D NOESY data (Supplementary Figure S2) show NOE cross-peaks between the benzene proton (H_a_) in entrapped **GA** and the protons H_1–4_ in **CP6**, indicating the inclusion of a benzene ring in the **CP6** cavity. The signals in the NMR spectra of **CA** and **FA** changed similarly upon the addition of **CP6** (Supplementary Figures S10,S11).

## Conclusions

To summarize, we demonstrated a new fluorescence activation switch based on host-guest complexation between the fluorescent indicator probe **TNS** and cationic pillar[6]arene **CP6**. In **TNS** solution, the complexation significantly enhanced the fluorescence. A fluorescence switch-off displacement assay was used to detect three commonly used non-fluorescence phenolic food additives in the water. The study used molecular recognition and fluorescence indicator displacement assays to develop a prospective strategy for phenolic food additive detection.

## Data Availability

The original contributions presented in the study are included in the article/Supplementary Material, further inquiries can be directed to the corresponding author.
