# Supplementary material for: The Detection of Food Additives Using a Fluorescence Indicator Based on 6– p–Toluidinylnaphthalence-2-sulfonate and Cationic Pillar[6]arene
Source: Front Chem. 2022 May 31;10:925881. doi: 10.3389/fchem.2022.925881 (PMC9194816; doi:10.3389/fchem.2022.925881)
Supplement: Supplementary file 1 [file Table1.DOCX]

Supplementary Material

1. *2D NOESY spectra of* ***CP6****⊃* ***TNS*** */****GA*** *complexes*


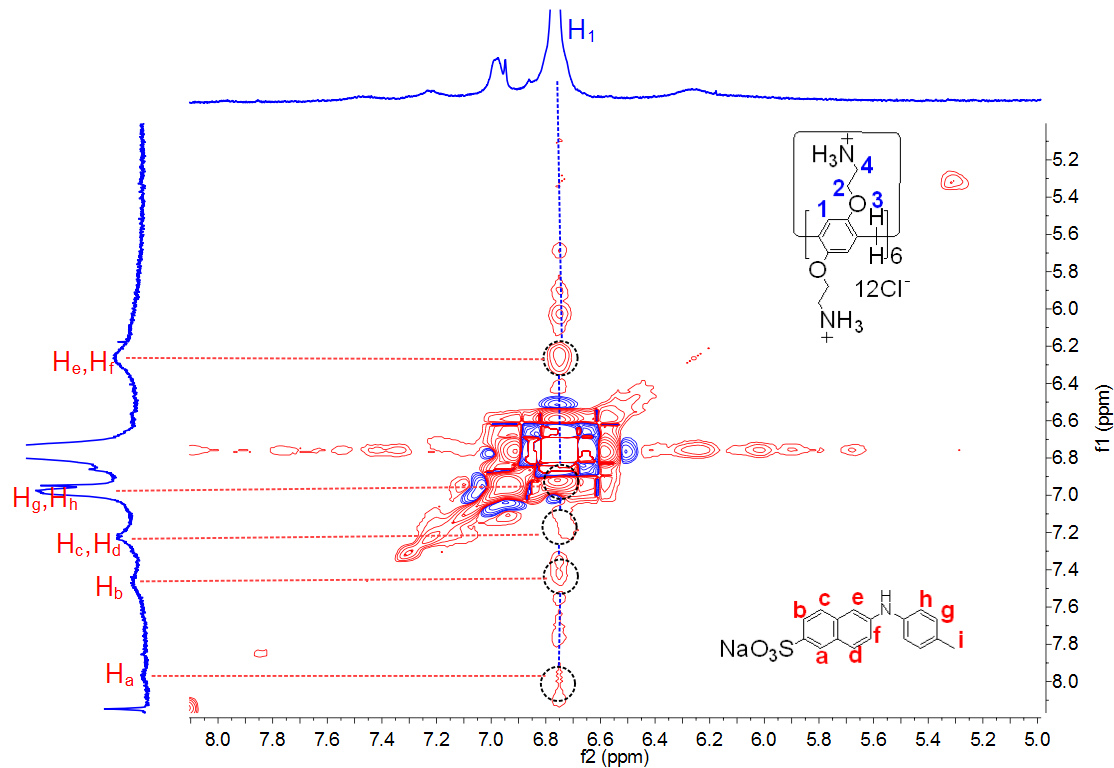


**Supplementary Figure 1.** 2D NOESY NMR spectrum of **CP6**⊃**TNS** (400 MHz, D_2_O-DMSO-*d*_6_ = 3:1, 298 K, mixing time = 300 ms), [**CP6**] = 5.0 mM, [**TNS**] = 15.0 mM.


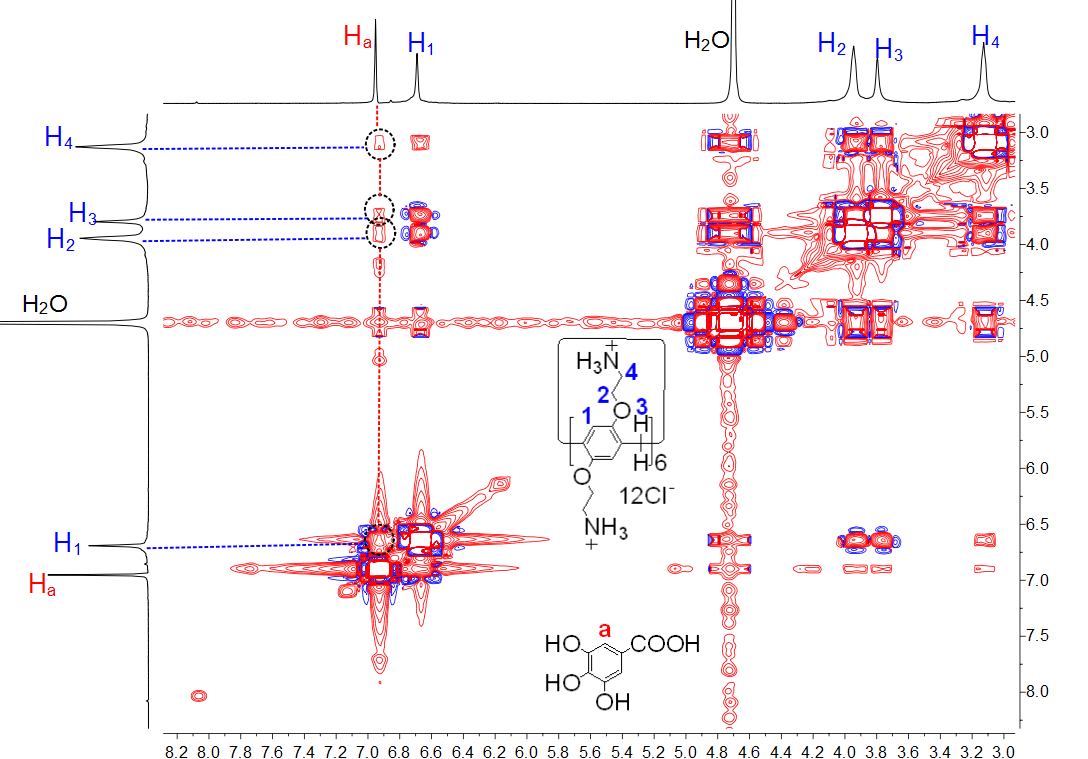


**Supplementary Figure 2.** 2D NOESY NMR spectrum of **CP6**⊃**GA** (400 MHz, D_2_O-DMSO-*d*_6_ = 3:1, 298 K, mixing time = 300 ms), [**CP6**] = 5.0 mM, [**GA**] = 15.0 mM.

*2. Determination of the association constants between substrates (****TNS****,* ***CA****,* ***FA, and GA****) and* ***CP6***

*2.1 Job plot for* ***CP6****⊃* ***TNS*** */* ***CA*** */* ***FA / GA***












**Supplementary Figure 3.** (a-d) Job plot showing the 1:1 stoichiometry of the complex between **CP6** and substrates (**TNS**, **CA**, **FA**, **GA**) by plotting the difference in fluorescent emission intensity at *λ*_em_ = 326 nm (*λ*_ex_ = 290 nm) against the mole fraction of substrates at an invariant total concentration of 0.01 mM in aqueous solution, respectively.

*2.2 Association constants of* ***CP6****⊃* ***TNS***







**Supplementary Figure 4.** (a) Fluorescence spectra of **TNS** (2.0 × 10^–5^ M) upon addition of **CP6** (0-5.2 × 10^–5^ M) in aqueous solution (excited at 318 nm) at room temperature. (b) The fluorescence intensity changes of **TNS** upon addition of **CP6**. The pink solid line was obtained from the non-linear curve-fitting ^S1^.

*2.3 Association constants of* ***CP6****⊃* ***CA/FA/GA***







**Supplementary Figure 5.** **Competitive fluorescence titration and the associated titration curve of CP6•TNS reporter pair and CA.** (a) Competitive fluorescence titration of CP6**•**TNS (0.48/0.2 μM) with CA (up to 2300 μM), *λ*_ex_ = 318 nm. (b) The associated titration curve at *λ*_em_ = 450 nm and fit according to a 1:1 competitive model.







**Supplementary Figure 6. Competitive fluorescence titration and the associated titration curve of CP6•TNS reporter pair and FA.** (a) Competitive fluorescence titration of CP6**•**TNS (0.48/0.2 μM) with FA (up to 900 μM), *λ*_ex_ = 318 nm. (b) The associated titration curve at *λ*_em_ = 450 nm and fit according to a 1:1 competitive model.







**Supplementary Figure 7. Competitive fluorescence titration and the associated titration curve of CP6•TNS reporter pair and GA.** (a) Competitive fluorescence titration of CP6**•**TNS (0.48/0.2 μM) with GA (up to 8900 μM), *λ*_ex_ = 318 nm. (b) The associated titration curve at *λ*_em_ = 450 nm and fit according to a 1:1 competitive model.










**Supplementary Figure 8.**  Calibration curves of fluorescence intensity of CP6**•**TNS reporter pair against CA (a), FA (b), and GA (c) concentrations in PBS buffer. F and F_0_ are the fluorescent intensities of the CP6**•**TNS reporter pair with and without the guest molecules, respectively. All experiments were performed at 25 oC, *λ*_ex_ = 318 nm, and *λ*_em_ = 450 nm. Error bars represent mean ± s.d. (n = 3 independent experiments).

*3 . ^1^H NMR spectra of substrates (****GA****,* ***CA****,* ***FA****) in the absence and presence of* ***CP6***


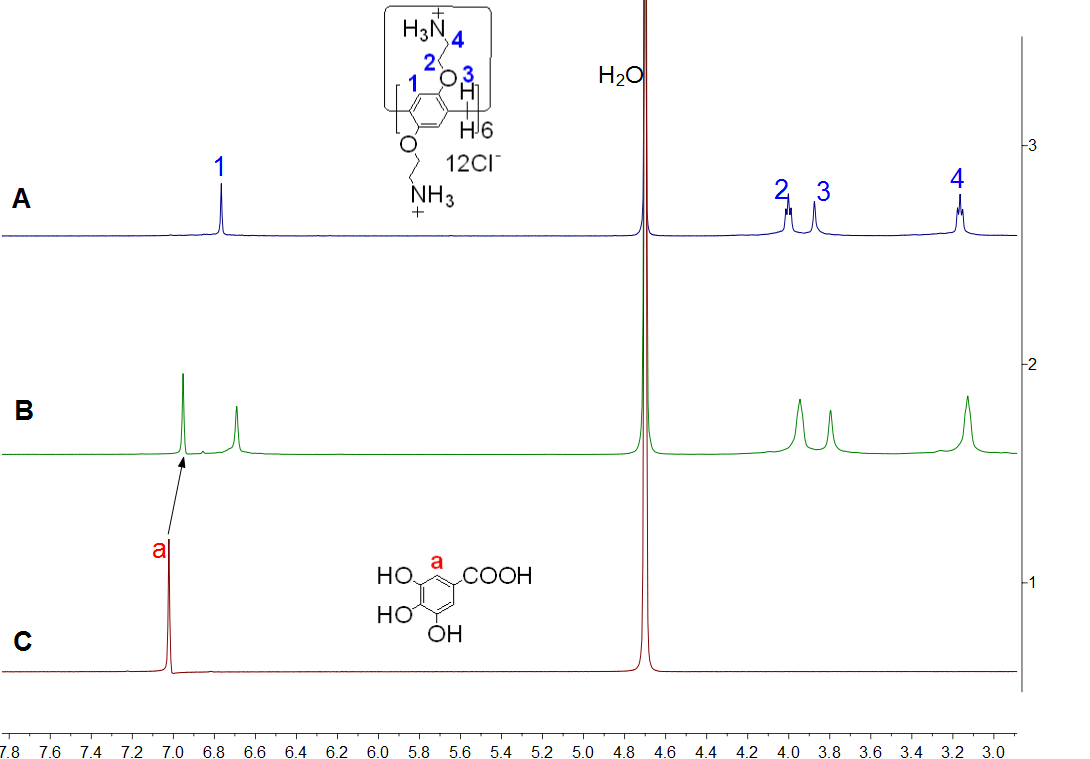


**Supplementary Figure 9.** ^1^H NMR spectra (400 MHz, D_2_O, 293 K) of (**A**) 5.0 mM **CP6**, (**B**) 5.0 mM **CP6** + 15.0 mM **GA**, and (**C**) 15.0 mM **GA**.


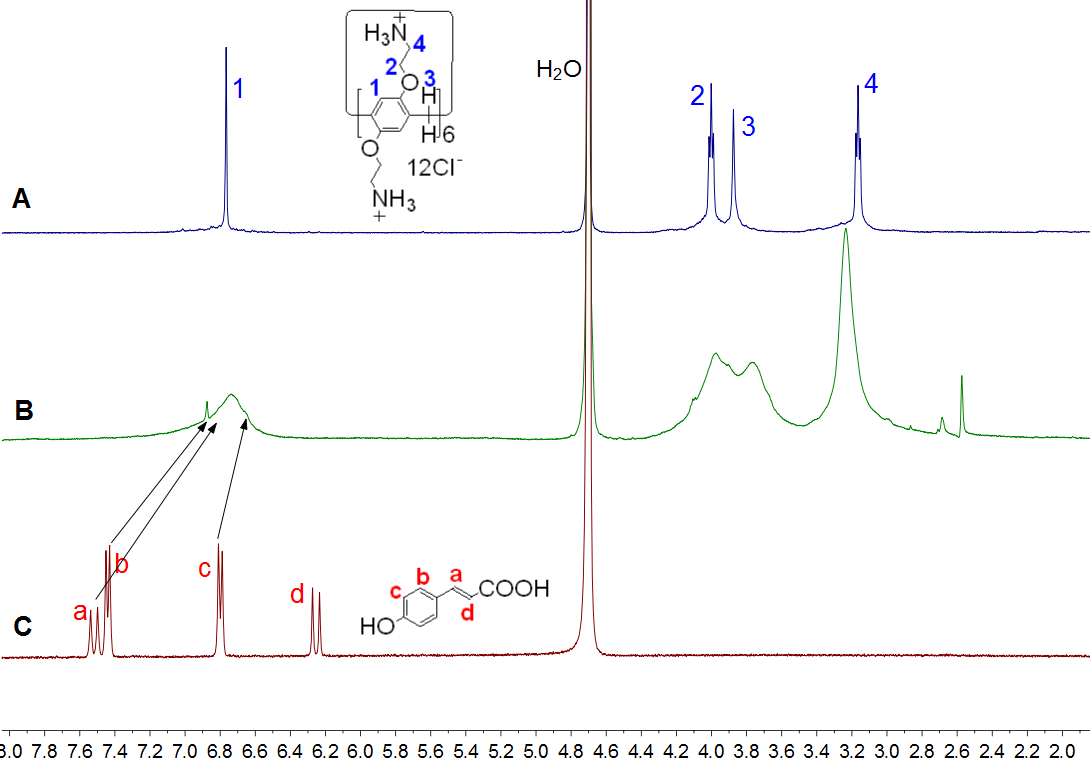


**Supplementary Figure 10.** ^1^H NMR spectra (400 MHz, D_2_O, 293 K) of (**A**) 5.0 mM **CP6**, (**B**) 5.0 mM **CP6** + 15.0 mM **CA**, and (**C**) 15.0 mM **CA**.


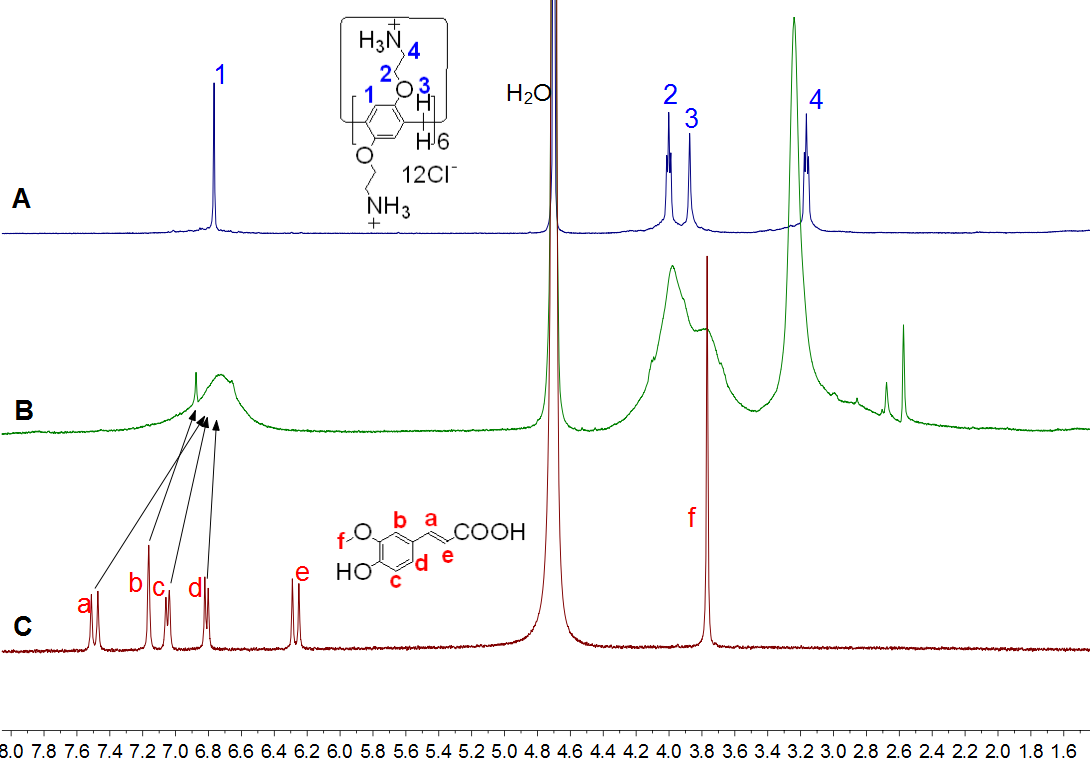


**Supplementary Figure 11.** ^1^H NMR spectra (400 MHz, D_2_O, 293 K) of (**A**) 5.0 mM **CP6**, (**B**) 5.0 mM **CP6** + 15.0 mM **FA**, and (**C**) 15.0 mM **FA**.

*References:*

- S1. (a) K. A. Connors, Binding Constants, Wiley: New York, 1987. (b) P. S. Corbin, Ph.D. *Dissertation*, University of Illinois at Urbana-Champaign, Urbana, IL, 1999. (c) P. R. Ashton, R. Ballardini, V. Balzani, M. Belohradsky, M. T. Gandolfi, D. Philp, L. Prodi, F. M. Raymo, M. V. Reddington, N. Spencer, J. F. Stoddart, M. Venturi, D. J. Williams, *J. Am. Chem. Soc.* 118 (1996) 4931−4951. (d) J. Zhang, F. Huang, N. Li, H. Wang, H. W. Gibson, P. Gantzel, A. L. Rheingold, *J. Org. Chem.* 72 (2007) 8935−8938.
